# Evaluating the Holistic Value of Family‐Centred Care in Paediatric Intensive Care Units: A Mixed Methods Study

**DOI:** 10.1002/nop2.70752

**Published:** 2026-10-21

**Authors:** Alhassan Sibdow Abukari, Shelley Schmollgruber, Emmanuel Kwame Korsah

**Affiliations:** ^1^ Department of Nursing Education, Faculty of Health Sciences University of the Witwatersrand Johannesburg South Africa; ^2^ Department of Maternal and Child Health, School of Nursing and Midwifery University of Ghana Accra Ghana; ^3^ Division of Clinical Associates, Department of Family Medicine and Primary Care, Faculty of Health Sciences University of the Witwatersrand Johannesburg South Africa

**Keywords:** FCC, holistic value of FCC, mixed‐methods approach, paediatric intensive care units

## Abstract

**Aim:**

This study aimed to explore the perceived holistic value of family‐centred care (FCC) in Ghanaian paediatric intensive care units (PICUs) from the perspectives of nurses, doctors, and families.

**Design:**

This is a four‐phase mixed‐methods study that incorporates literature control, qualitative, instrument development, and quantitative survey.

**Methods:**

The researchers purposely conducted 12 individual interviews with six PICUs nurses and doctors (three nurses and three doctors) and six families (three fathers and three mothers) with sick children in two PICUs. Thematic analysis was employed to manage the qualitative data. Qualitative findings were used to develop a questionnaire for an electronic survey (*n* = 100), and the data were analysed using exploratory factor analysis, Principal Component Analysis, and commonalities.

**Results:**

The study demonstrates the perceived holistic value of FCC in promoting family‐child bonding, reducing family anxiety, reducing workload, promoting recovery, providing timely information, and educating families on childcare processes in intensive care units. Most respondents (95%) agreed with the identified FCC value, indicating broad acceptance. The internal consistency of the holistic value of FCC, as measured by the Cronbach alpha coefficient (> 0.82), further validated the results. The primary contextual value identified by Principal Component Analysis was the FCCs role in promoting family education, with a significant Eigenvalue of 3.200 and accounting for 53.337% of the cumulative variance.

**Conclusion:**

FCC practices empower families to continue providing appropriate childcare after discharge, which has been perceived as reducing anxiety, promoting bonding, and fostering good parenting for critically ill children. Participants perceived a high level of agreement that the FCC also shortens hospital stays and reduces workload. To improve the quality of paediatric care in resource‐constrained settings, healthcare professionals and policymakers should prioritise implementing FCC practices.

**Implication for Practice:**

Our findings suggest that FCC integration into PICU could improve quality health outcomes, particularly for children and their families. As such, nurses and doctors may require additional in‐service training on FCC integration into the PICU setting. It is proposed that heads of PICUs could modify the set‐up to create space for easy FCC integration.

**Reporting Method:**

This study is reported using Mixed Methods Reporting in Rehabilitation and Health Sciences.

**Patient or Public Contribution:**

No patient or public contribution.

**Clinical Trial Number:**

The authors have nothing to report.

## 1 Introduction

Family‐centered care (FCC) is an evidence‐based, humanistic approach that includes families as vital partners in the care of critically ill children. Beyond clinical outcomes, FCC provides holistic value by promoting emotional well‐being, improving communication, reducing provider burnout, and ensuring culturally appropriate care (Chung and Chae 2023; Wickramasinghe et al. 2019). While the impact of the FCC in adult intensive care settings is well documented, its holistic value in paediatric intensive care units (PICUs), particularly in low‐resource settings such as Ghana, remains underexplored. Using a mixed‐methods design, this study describes the perceived holistic value of FCC in Ghanaian PICUs from the perspectives of nurses, doctors, and family members. For the healthcare systems to foster trust, improve care continuity, and optimise outcomes, there is the need to incorporate FCC principles into policy, nursing education, and PICU protocols. While healthcare professionals often recognise the principles and benefits of the FCC, implementation may be hindered by contextual barriers, including staffing shortages, workload demands, infrastructure limitations, and organisational culture. This study emphasises the importance of FCC as a transformative strategy for improving the quality of paediatric care in Ghana and other resource‐constrained settings.

## 2 Background

FCC has attained increasing global recognition as an essential framework for improving health outcomes in paediatric and intensive care settings (Abukari and Schmollgruber 2025). FCC embodies a healthcare philosophy and methodology that positions the child and their family at the core of the care framework, acknowledging them as essential participants in clinical decision‐making and treatment planning (Abukari and Schmollgruber 2025; Ohene et al. 2019). This approach is grounded in principles of respect, dignity, and collaboration, underscoring the pivotal role of families in formulating, implementing, and evaluating healthcare (Abukari and Schmollgruber 2023). The holistic value of FCC has notably intensified within PICUs, where critically ill children rely not only on clinical interventions but also on the emotional support, bonding, and continuity of care families provide. The holistic value of FCC refers to the perceived cultural, psychosocial, relational, organisational and educational benefits associated with FCC, and not merely the traditional clinical outcome. Consequently, FCC has emerged as a vital mechanism in ensuring comprehensive paediatric health, particularly for vulnerable children in intensive care (Abukari and Schmollgruber 2025).

Historically, paediatric healthcare practices have been predominantly orientated around a biomedical model characterised by care led by experts with minimal family involvement (Abukari and Schmollgruber 2024; Kiwanuka et al. 2019). Nonetheless, recent evidence indicates that this model may inadequately address families' psychosocial needs and fail to acknowledge their critical role in caregiving, particularly in the PICU context (Abukari and Schmollgruber 2024). Numerous international studies emphasise the holistic value of FCC, including reduced child stress, shorter hospital stays, increased weight gain, improved breastfeeding outcomes, and enhanced family satisfaction (Dall'Oglio et al. 2019; Fotteler et al. 2022). A study in the Netherlands found that 7 of 17 intervention studies demonstrated significant reductions in ICU stay duration due to effective implementation of FCC (Oude Maatman et al. 2020). Moreover, families involved in FCC practices reported higher satisfaction levels and emotional well‐being, underscoring the transformative potential of FCC beyond merely medical outcomes (Oude Maatman et al. 2020).

Despite documented holistic value, significant disparities persist in implementing FCC, particularly in low‐ and middle‐income countries (LMICs), where health systems face diverse structural and cultural challenges (Abukari and Schmollgruber 2024). In several African and West African contexts, FCC remains inadequately defined or underutilised, frequently impeded by insufficient staffing, inadequate infrastructure, and the lack of institutional guidelines (Abukari and Schmollgruber 2024; Nkrumah and Abekah‐Nkrumah 2019). In Ghana, for instance, although families play an essential role in hospitalisation, providing financial support, emotional sustenance, and basic caregiving, formalised FCC frameworks are rarely institutionalised (Abukari et al. 2022). Family involvement tends to be informal and reactive instead of structured and policy driven. Cultural norms also distinctly influence FCC practices within this context, where extended family systems, including grandparents, siblings, and community members, often contribute to paediatric care (Abukari et al. 2022; Abukari and Schmollgruber 2025). Nevertheless, this abundant resource remains underutilised due to the absence of structured engagement and clinician orientation.

A significant concern is the persistent uncertainty among nurses and doctors and healthcare administrators about the holistic value of FCC in the PICU. For instance, some professionals recognise the essential nature of FCCs, yet others question their practicality due to the considerable workloads, infection risks, and logistical constraints intrinsic to ICUs (Kim 2020; Moore et al. 2015). Furthermore, the spatial configuration of PICUs in numerous Ghanaian hospitals, often characterised by limited space, overcrowding, and a lack of privacy, further intensifies the challenges of incorporating FCC into care protocols (Abukari 2022; Abukari and Schmollgruber 2024). These infrastructural limitations often necessitate restrictive visitation policies, limiting family engagement in critical care decisions. Consequently, although parents are afforded limited access to their children in Ghanaian PICUs, the holistic value of FCC remains largely unexamined or inconsistently applied.

The lack of empirical evidence on the holistic value of FCC within the Ghanaian healthcare system undermines advocacy efforts to implement it widely. While FCC practices are increasingly incorporated into policy discussions and selected clinical settings, there is a scarcity of rigorous research assessing their holistic value in Ghana. This scarcity of evidence hinders the development of national guidelines, training programs, and institutional protocols that are essential for integrating FCC into paediatric intensive care. A comprehensive examination of the perspectives of both families and healthcare practitioners within this context is crucial to expose the holistic value of FCC. The need to address this gap is further underscored by the high paediatric mortality rates in sub‐Saharan Africa, where family involvement may play a crucial role in improving outcomes (Mohammadi et al. 2021; Mohammed and Tak 2022). Therefore, this study aligns with national and international priorities to improve paediatric survival rates and promote family involvement in child healthcare. This research could contribute to the broader discourse on implementing FCC in LMICs, as it was executed in similar resource‐limited settings. Consequently, this study aims to evaluate the holistic value of FCC in Ghanaian PICUs from the perspectives of nurses, doctors, and families.

## 3 Methods

### 3.1 Study Design

We used a four‐phase mixed‐methods approach to evaluate the holistic value of FCC in Ghanaian PICUs (Gaglio et al. 2020). This study adopted a qualitative, exploratory, descriptive design, as it accommodates a pragmatic research approach (Pelzang and Hutchinson 2018). The qualitative exploratory descriptive (QED) design was used to gain insight into participants' ontological and epistemological perspectives on the holistic value of FCC (Pelzang and Hutchinson 2018). Firstly, a literature control was conducted, and the findings were used to develop a semi‐structured interview guide. In the second phase, a qualitative exploration was conducted to explore the perceived holistic value of FCC among families, nurses, and doctors in PICU. In phase three, the qualitative findings were triangulated and mapped to develop a four‐point Likert scale survey instrument for the quantitative study. The fourth phase was a descriptive quantitative study among PICU staff (nurses and doctors), and the data were exposed to exploratory principal component analysis to obtain a succinct outcome for confirmation and generalisation (Harerimana and Mtshali 2020). The quantitative analysis was conducted as an exploratory principal component analysis (PCA) to examine the underlying structure of the perceived holistic value of the FCC and not a scale development. The study is reported in line with Mixed Methods Reporting in Rehabilitation and Health Sciences (MMR‐RHS). Refer to Figure 1 for the methodological phases of the study.

**FIGURE 1:**
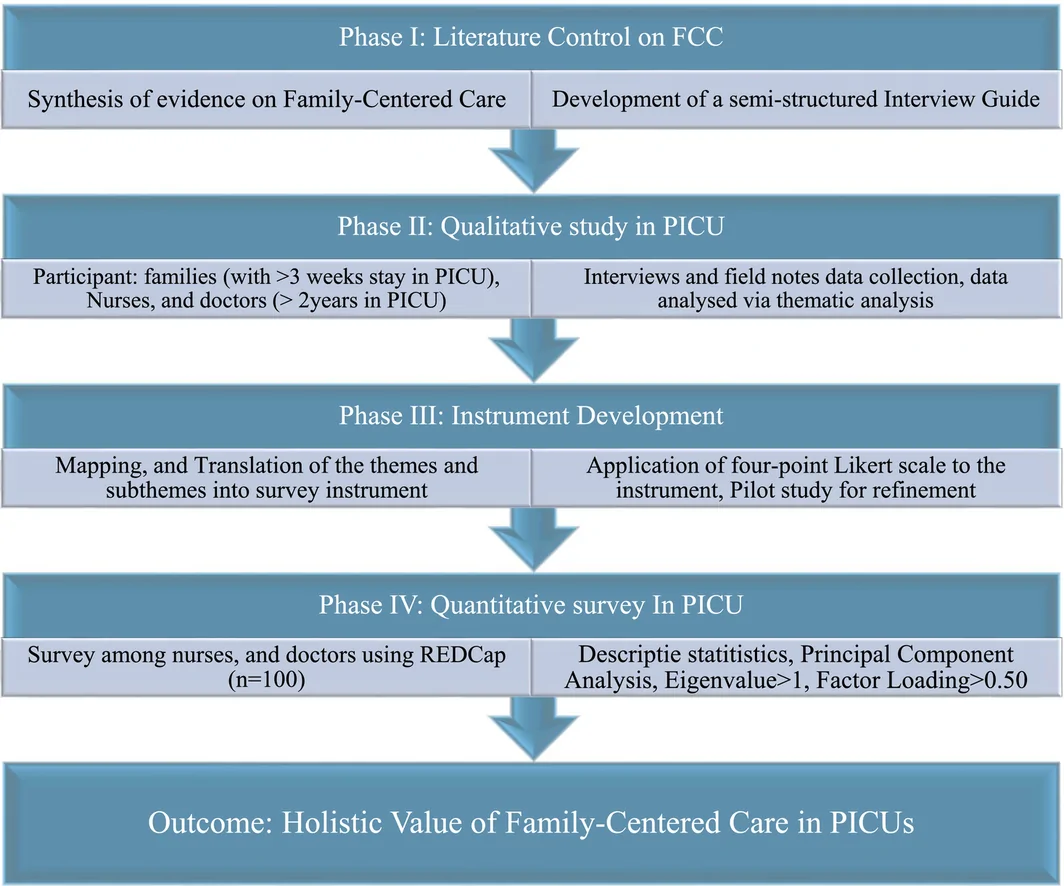
Methodological phases of the study.

### 3.2 Setting

The research was conducted in two public tertiary PICUs. These ICUs provide a range of medical‐surgical conditions, including aspiration pneumonia and paediatric emergency traumas. The ICUs serve as referral centres with adequate ICU equipment, including ventilators, cardiac monitors, and electronic vital sign monitors. The clinical staff includes specialists such as paediatric nurses, intensive care nurses, paediatricians, intensive care physicians, and dieticians.

### 3.3 Study Participants and Sampling

The study adopted purposive sampling to select participants with relevant information on the topic. This study explicitly recruited PICU staff, including nurses and doctors with over 2 years of working experience. Participants who also met the inclusion criteria were families with critically ill children admitted to the PICU for at least 3 weeks. The qualitative participants differed from those recruited for the quantitative study to ensure data variability.

### 3.4 Data Collection Procedures

Interviews were used to gather qualitative data, and surveys were used to collect quantitative data. The family interviews were held independently of the clinician interviews, although all were conducted in the same PICU setting using researcher‐developed interview guides based on objectives and literature review. Participants who met the inclusion criteria were recruited for the interviews by the first author, with support from the unit in charge. Participants were given an information leaflet, and those who volunteered to participate completed an informed consent form before data collection. Additional permission to record audio was obtained from all qualitative participants. All interviews were audiotaped in a secure room to exclude distractions such as noise and to maintain privacy and confidentiality. Each interview lasted roughly 45 min. The first author conducted six interviews in each PICU: two nurses, two doctors, and two family members (father and mother). Additionally, field notes were taken to support the interview data. The qualitative findings were synthesised into a survey questionnaire with a Likert scale ranging from 1 to 4: 1 = Strongly disagree; 2 = Disagree; 3 = Agree; and 4 = Strongly agree. An aggregate score of 1 and 2 = Negative practice; that of 3 and 4 = Positive practice. Before the quantitative survey, the instrument was pretested involving different samples from the same study population to add data variability and confirm the findings. A purposive sampling strategy was used to recruit PICU staff via an electronic RedCap survey link (*n* = 100). The first author, a native male nurse scientist, specialised in paediatric care, managed the data.

### 3.5 Data Analysis

The audiotapes were transcribed verbatim into English text as data transcripts. Field notes were added to the transcripts during the data collection to enrich the data. The transcripts and the synthesised findings were returned to some participants for verification. We employed thematic analysis (TA) to deductively and inductively analyse the data to find shared meaning across datasets (Kiger and Varpio 2020). Familiarisation, code creation, searching for themes, assessing themes, defining and naming themes, and report writing are the six processes employed in the thematic analysis of qualitative data (Kiger and Varpio 2020). Two themes were observed: the family value of FCC and the facility value of FCC. Initially, the researchers reviewed the transcripts to correct grammatical errors and familiarise themselves with the data. Codes were then assigned to relevant concepts in each transcript. The generated codes were then used to search each transcript in MAXQDA, and the codes were merged into subthemes, which were reviewed and aggregated into findings.

For the quantitative data, a Principal Component Analysis (PCA) was performed using SPSS v.29 using Varimax Orthogonal Rotation. The criteria for retaining variables were based on the Eigenvalue greater than one rule, commonalities, and factor loading above 0.50 (Shrestha 2021).

### 3.6 Methodological Rigour

This study's data collection and analysis techniques were iterative to achieve rigour. To generate a contextual finding, we engaged a multidisciplinary group of participants, including nurses, doctors, and family members. The prepared interview guide was developed using findings from a literature review to ensure validity. We provided the participants with an information sheet before the study to elicit objective assessments. Finally, validity and reliability estimates, such as Cronbach's alpha, the Kaiser–Meyer–Olkin (KMO) sample adequacy score, and Bartlett's test of Sphericity, were conducted, which showed that the data were adequate for factor analysis (Harerimana and Mtshali 2020).

### 3.7 Ethical Considerations

We obtained ethical approval for the study from the appropriate the two local facilities and university ethics committees. The initial ethics was approved in 2019, and an extension was granted in 2022. Additionally, we provided an information sheet to all participants, and those who agreed to participate signed a voluntary consent form before the study. Additional consent for voice recording was voluntarily obtained from the participants. We used the introductory letters from the ethics committee to access the department and the facilities for data collection. Also, we followed all ethical guidelines on confidentiality, autonomy, and voluntary participation as enshrined in the Helsinki Declaration (Ehni and Wiesing 2024).

## 4 Results

### 4.1 Qualitative Results

#### 4.1.1 Perceived Holistic Value of FCC

This refers to the physical, social, cultural, and emotional value of FCC in the PICU from the perspectives of nurses, doctors, and families. Two themes emerged from the data: the perceived family value of FCC and the perceived facility value of FCC. Various subthemes were aggregated from the participants' perspectives to support these themes. Refer to Table 1 for the themes and subthemes.

**TABLE 1:** Themes and subthemes.

Theme	Subtheme
1. Perceived family value of FCC	FCC promotes family participation and education on childcare
	FCC promotes continuity of care and rehabilitation
	FCC promotes family cooperation and confidence in paediatric care
	FCC promotes a triangle of bonding between clinicians, family, and the child
	FCC has perceived effects on managing family psychological stress during child illness
	FCC perceived effects on shortening the length of hospitalisation of critically ill children
2. Perceived facility value of FCC	FCC promotes the best outcome for early discharge of critically ill child
	FCC reduces paediatric and child mortality in the ICU
	FCC reduces workload in the ICU
	Improves information sharing to aid decision‐making

##### 4.1.1.1 Theme 1: Perceived Family Value of FCC

This theme refers to the specific value of FCC to the family. Six subthemes were aggregated to support this theme.

###### 4.1.1.1.1 Subtheme 1: FCC Promotes Family Participation and Education on Childcare

Nurses and doctors and families emphasised the value of FCC in Ghana's PICUs for promoting family participation, educating on childcare, fostering empowerment, and strong family‐child bonding.

‘Initially, I had challenges, but the staff taught me how to feed, and now I am experienced in feeding comfortably…’ KFI2, Mother 2.

‘We educate the family on their child's condition and the care we are giving; it helps the family to prepare their mind about the outcome of the care process.’ TSI6: Paediatrician 1.

###### 4.1.1.1.2 Subtheme 2: FCC Promotes Continuity of Care and Rehabilitation

Nurses and doctors perceived that FCC's transformative approach could be valuable in home care and rehabilitation, encouraging active family participation in nursing duties, instilling confidence, and fostering acceptance and positive morale, especially for children with congenital anomalies.

‘The advantage is that once they stay in the ward with the children and participate in some care, they start practicing before they go home…practice better home care on discharge’, TPI1, Paediatrician 1.

‘The family involvement and participation will help us to continue the care at home…’ KFI5, father 2.

‘It promotes rehabilitation, especially for those with congenital anomalies, as the family will accept those children and accept them as part of their family…’ Paediatric nurse.

###### 4.1.1.1.3 Subtheme 3: FCC Promotes Family Cooperation and Confidence in Paediatric Care

Paedestrians largely perceived FCC to foster family cooperation and confidence in childcare within the PICU through continuous updates, active involvement, and transparent communication.

‘: … I have realised that parents who are updated and involved in the care of their children are very cooperative’ KPI5, Paediatrician 5.

‘…it makes the family feel part of the care rendered to their child, and this boosts their confidence to care for the child at home after discharge’ TCD1C, Paediatric Nurse 3.

‘… my child's respiration was bad; they placed him under some machine. Honestly, the way the nurse explained the care … is refreshing, and that has boosted my confidence’. TFI3, mother 2.

###### 4.1.1.1.4 Subtheme 4: FCC Promotes a Triangle of Bonding Between Nurses and Doctors, Family, and the Child

FCC fosters a dynamic bonding triangle in the PICU environment, promoting collaboration among healthcare professionals, families, and children despite challenges faced by some families, and emphasising the importance of consistent implementation of FCC.

child ‘: …allowing the family into the ICU, especially the father and the mother, to be part of the management builds that bond…’ KPI4, Paediatrician 4.

‘: ‘…my child was crying, and the staff called me; immediately, I talked, and the child stopped.

crying, so I realised there was some bond…’ KFD1B mother 2.

‘Bringing the family closer, especially the mothers, is very important as it will help promote bonding with children’, TSI3, Nurse Intensivist.

###### 4.1.1.1.5 Subtheme 5: FCC Has Perceived Effects on Managing Family Psychological Stress During Child Illness

FCC could enhance the well‐being of families with children in the PICU by reducing stress, anxiety, and psychological stress associated with the ICU environment.

‘So, I do not see child health as something you can do without the family. If you do not involve them, the child will stay longer, and the family will get frustrated because they have left their work. All these add up to the family stress and anxiety…’ KPI2, Paediatrician 2.

‘FCC makes them feel part of the treatment process and reduces the psychological trauma to the family’, TCD1A Registered Nurse.

###### 4.1.1.1.6 Subtheme 6: FCC Perceived Effects on Shortening the Length of Hospitalisation of Critically Ill Children

The FCC in Ghana's PICUs is perceived to accelerate critically ill children's recovery, shorten hospitalisations, and promote early discharge.

‘…it would help in the speedy recovery and, in effect, limit the length of stay of the children in the ICU’, TCI2, Medical doctor.

‘It reduces the length of stay as the recovery rate will be faster. Hence, *nurses and doctors* should be motivated to practice FCC’ TCD1C, Registered Nurse.

‘Some quality care can be rendered to the patient because the health team and the family are all immediately targeting the child's discharge. In our setting, early discharge means you have done a good job…’ TCI5, father 1.

##### 4.1.1.2 Theme 2: Perceived Facility Value of FCC

This theme highlights the FCC's importance to the facility. Four subthemes were aggregated to support this theme.

###### 4.1.1.2.1 Subtheme 1: FCC Promotes the Best Outcome for Early Discharge of the Critically Ill Child

This study demonstrates the perceived value of the FCC in achieving optimal health outcomes for critically ill children. Participants emphasised collaborative healthcare and informed decision‐making to maximise its value.

‘I think working together will bring out the best outcome for the child…FCC is a good thing to implement in the ICU’ KPI2, Paediatrician 2.

‘One thing I like about the staff is that before they give any treatment… they conduct a series of lab tests, and I think it helped my child to get the right treatment and recover’, KFI3, mother 2.

###### 4.1.1.2.2 Subtheme 2: FCC Reduces Paediatric and Child Mortality in the ICU

According to the participants, involving families in the care process improves the home‐care transition, reducing the likelihood of mortality.

‘It further reduces paediatric mortality rates as families are involved in the care, and they can continue the care at home’, TCD1AD, Registered Nurse.

‘It will reduce child mortality if the education goes down well…’ KFD1A, father 1.

‘…family inclusion in the care of these sick children will promote their recovery and help reduce mortality…’ TCD5, Medical doctor.

###### 4.1.1.2.3 Subtheme 3: FCC Reduces Workload in the ICU

The study highlights the perceived positive impact of FCC on reducing workload in Ghanaian PICUs, emphasising the importance of involving families, particularly mothers, in childcare, thereby reducing the workload on healthcare professionals and improving overall PICU efficiency and care quality.

*‘*Family assists the nurse in caring for the children, especially the feeding…it can reduce workload’, KSD1C, Paediatric nurse.

*‘*Our presence in the ICU will assist the nurses in caring for the children by feeding them, cleaning them, changing diapers and clothing, and notifying the *nurses and doctors*.of any sudden changes in the children's conditions’ TFI4, mother 1

‘The work overload will reduce if mothers are involved’, KPI2, Paediatrician 2.

###### 4.1.1.2.4 Subtheme 4: Improves Information Sharing to Aid Decision‐Making

FCC fosters a collaborative environment, promotes transparency and information sharing, empowers families in healthcare decisions, enhances overall paediatric care, and improves communication among paediatricians and families.

‘…Family‐centered care can help us because the mother would give all the information you need about the child to make decisions regarding the care you give to them…’ TCI2, Paediatric nurse.

‘FCC sets up and clarifies the whole process of care for the appreciation of the families, and that is one very important issue about having the family participate in the ICU’, TPI1, Paediatrician 1.

‘They usually educate us; education is very key in this whole issue, …to know the management giving to the children …’ KFD1, father 2.

### 4.2 Quantitative Results

#### 4.2.1 Relevant Biographical Details

Twelve participants were recruited qualitatively, and 100 respondents participated in the quantitative survey (> 95% response rate). The PICU staff had an average of 5 years of experience. Table 2 provides more information about the respondents in the quantitative study. The average age of the PICU staff was 31.93 ± 5.204 years, with a minimum age of 23 years and a maximum age of 53 years, with females accounting for the majority (69%; *n* = 69). The nurses and doctors had an average of 5.67 years of clinical experience, ranging from 2 to 28 years. Most PICU staff (63%) had less than 5 years of experience. Most PICU staff (81%) was nurses, with the remaining 19% working in medicine, midwifery, and nutrition. Many PICU staff (58%) is generalists working in intensive care. Only 7% of the respondents were paediatricians, 4% were paediatric intensive care nurses, and 8% were paediatric nurses. For more information, see Table 2, demographics of the respondents.

**TABLE 2:** PICU staff’ demographic data (*N* = 100).

	Frequency (*N*)	Percentage (%)
**Demographic variables**		
Age		
20–29	34	34
30–39	54	54
40–49	11	11
> 49	1	1
Gender		
Male	31	31
Female	69	69
Religion		
Christian	70	70
Moslem	30	30
Marital status		
Single	40	40
Married	60	60
**Professional variables**		
Educational qualification		
Certificate	8	8
Diploma	46	46
Degree	35	35
Masters	5	5
Doctoral	6	6
Profession		
Nursing	81	81
Midwifery	8	8
Medicine	10	10
Nutrition	1	1
Specialisation		
Paediatrician	7	7
Paediatric intensive care nurse	4	4
Paediatric nurse	8	8
Midwife	17	17
Ophthalmic nurse	2	2
ENT Nurse	2	2
Nutritionist	1	1
Physician assistant	1	1
No specialty	58	58
Years of practice		
1–5	63	63
6–10	27	27
11–15	7	7
16–20	2	2
> 20	1	1

#### 4.2.2 Descriptive Statistics

The benefit of the FCC scale is made up of six factors. The respondents had a favourable impression of the FCC's value with a degree of agreement: ≥ 3 for each item in the scale. Most respondents (≥ 95%) agreed with the items in the FCC Value, indicating favourable experiences. The Cronbach alpha test was used to analyse the internal consistency of the items on this scale, and the results showed that the items comprising the FCC value had high internal consistency (Cronbach alpha coefficient > 0.82). Overall, the scale had a standard deviation of 0.49 and an interquartile range of 3–4.

#### 4.2.3 Exploratory Factor Analysis: Principal Component Analysis and Communalities

Before factor analysis (FA), the data were examined for factorability, which typically involves normally distributed data and a sample size of at least 100 (Harerimana and Mtshali 2020). This quantitative stage has a sample size of 100. Furthermore, for the dataset to be considered suitable for FA, the Kaiser–Meyer–Olkin (KMO) measure of sampling adequacy index must be greater than 0.65 (Harerimana and Mtshali 2020). In the present study, Cronbach's α = 0.82, KMO = 0.85, and *p* < 0.001, indicating factorability.

PCA components were used to analyse the dataset further. Communalities show the percentage of variance explained by the collected components, and they are similar to regression. Normally, item variance with low communalities depicts unrelated concepts and vice versa (Balasundaram 2009; Gibbons 2016; Harerimana and Mtshali 2020; Jöresko 2015; Mahmoudi et al. 2020; Tripathi and Singal 2019). As a result, the higher the correlation between the defined characteristics and the core concept, the closer the factor loadings for the commonalities are to 1. The results of the PCA and the communalities are shown in Table 2. In the exploratory PCA, only one component was retained as shown in the scree plot (Figure 2). In the Ghanaian context, the perceived priority values of FCC are promoting bonding, early recovery, timely information sharing, and teaching families about childcare, as shown in Table 3. From Table 3, the mean scores indicated strong agreement (94%–100%) demonstrating that FCC has a perceived value to enhance education, reduce anxiety, promote bonding, support recovery, provide resources, and reduce workload.

**FIGURE 2:**
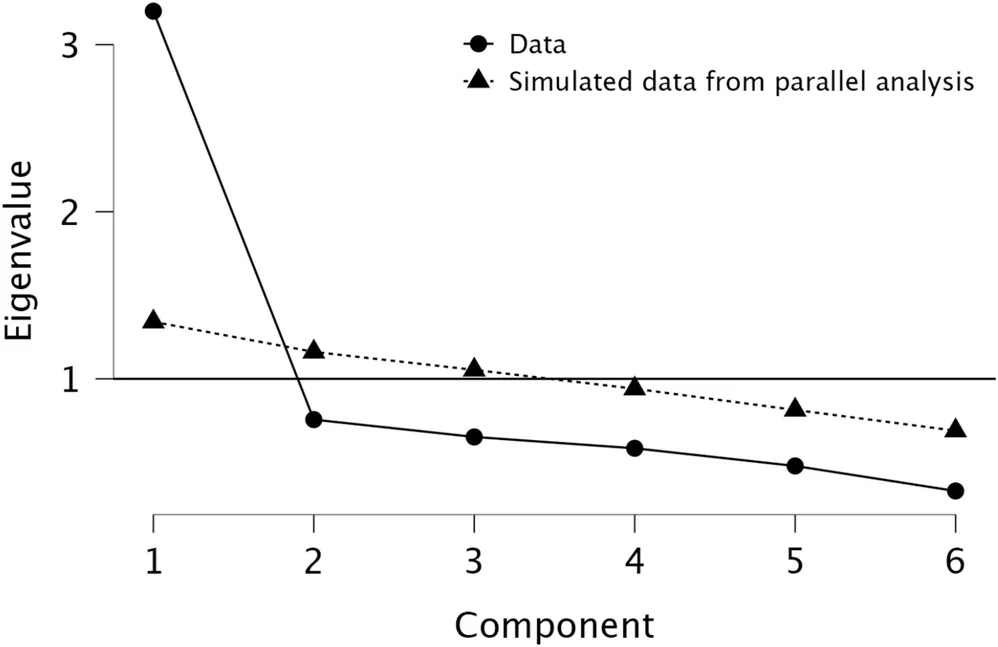
Scree Plot showing one component retention.

**TABLE 3:** Item‐level statistics on the holistic value of FCC.

Value of FCC (6 items)	Mean	SD	IQR	%Agree	%Strongly agree	%Total agreement
FCC practices serve as education for families especially new families to empower them to continue appropriate childcare at home after discharge	3.460	0.501	1	54.5	45.5	100
FCC practices reduce anxiety and motivate psychologically stressed families to support/participate in the care of critically ill children	3.380	0.488	1	61.4	37.6	99
FCC practices promote bonding and initiate good parenting for critically ill children	3.390	0.490	1	60.4	38.6	99
FCC practices promote early recovery and shorten the length of hospitalisation of children and families at the intensive care	3.300	0.595	1	58.4	35.6	94
FCC play significant roles in providing timely information and resources to support the clinical care of critically ill children	3.270	0.510	1	66.3	29.7	96
FCC practices reduce workload as they are well involved in the care processes	3.310	0.581	1	59.4	35.6	95

*Note:* %Agree represents the proportion of participants selecting ‘Agree,’ %Strongly Agree represents the proportion selecting “Strongly Agree,” and %Total Agreement represents the combined percentage of participants selecting ‘Agree’ or ‘Strongly Agree.’ Higher values indicate greater consensus among participants. IQR ≤ 1.0 and total agreement ≥ 75% were considered indicators of consensus.

Abbreviations: IQR, interquartile range; Mean, average item score; SD, standard deviation.

According to Table 4, the total variance explained for the value of FCC following the exploratory PCA was for educated families, with an Eigenvalue of 3.200% and 53.337% cumulative variance explained. The retained items included FCC is perceived to educate families (loading = 0.744), FCC is perceived to reduce anxiety (loading = 0.773), FCC is perceived to promote bonding (loading = 0.832), FCC is perceived to promote recovery (loading = 0.728), FCC is perceived to provide timely information (loading = 0.665), and FCC is perceived to reduce workload (loading = 0.619). These estimates indicate adequate representation of the items within the component. Sampling adequacy was acceptable, with a Kaiser–Meyer–Olkin (KMO) value of 0.845, while Bartlett's test of sphericity was significant (*χ* ^2^ = 187.585, df = 15, *p* < 0.001), supporting the suitability of the data for factor analysis. The extracted component had an eigenvalue of 3.200, confirming a unidimensional structure for the FCC value construct in PICUs. Refer to Table 4 for details.

**TABLE 4:** Exploratory principal component analysis of FCC value in PICUs.

FCC dimension/concept							Component 1 retained: FCC educate families
	Defined characteristics	Initial	Extraction	MSA	Real data component eigenvalues	Simulated data mean eigenvalues	
Education	FCC is perceived to educate families	1	**0.744**	0.869	3.200	1.319	**Retained**
Psychological support	FCC is perceived to reduce anxiety	1	**0.773**	0.816	0.756	1.168	
Psychological support	FCC is perceived to promote bonding	1	**0.832**	0.808	0.652	1.046	
Partnership	FCC is perceived to promote recovery	1	**0.728**	0.849	0.584	0.949	
Information sharing	FCC is perceived to provide timely information	1	0.665	0.897	0.479	0.822	
Family involvement	FCC is perceived to reduce workload	1	0.619	0.877	0.329	0.697	
**Model statistics**	*Eigen values*						3.200
	Cronbach alpha						0.820
	*Overall KMO*						0.845
	*Bartlett's χ* ^*2*^ *(df = 15)*						187.585
	*Bartlett's* *p*‐value						< 0.001
	*Model χ* ^*2*^ *(df = 9)*						16.832
	*Model* *p*‐value						0.001
	*The proportion of variance (%)*						53.337
	*Cumulative variance explained (%)*						**53.337**

*Note:* PCA was conducted to examine the communalities of items representing the ‘Value of Family‐Centred Care (FCC)’ construct. Initial communalities were set at 1.0, indicating that all variance in each item was assumed to be common prior to extraction. Extraction communalities represent the proportion of variance in each item explained by the retained component (0.50). A single factor was retained based on the Kaiser criterion (Eigenvalue = 3.200), which explained 53.337% of the total variance, indicating substantial shared variance among the items. Higher extraction values reflect stronger representation of the item within the underlying construct. Items with moderate communalities were retained based on theoretical relevance to FCC dimensions, including information sharing, psychological support, partnership, and family involvement. The bold values are the extracted perceived holistic values of FCC.  Statistical significance was set at *p* < 0.05.

## 5 Discussion

This mixed‐methods study in Ghanaian PICUs illuminates the perceived holistic value of FCC from the perspectives of families, nurses and doctors. We found that teaching families about childcare and providing timely information to families in PICUs have a holistic value in promoting recovery, early discharge, reducing family anxiety, and promoting bonding. The integration of FCC in PICUs could address mothers' initial emotional challenges related to the admission of their sick children in PICUs, thereby promoting a bond between families and their children (Hodgson et al. 2024; Latour et al. 2024). Additionally, the FCC also promotes continuity of care and rehabilitation, laying the groundwork for a smooth home‐care transition (Dall'Oglio et al. 2019; Yangjin et al. 2025). It also reduces stress and anxiety, improving parents' overall mental health, particularly nursing mothers (Zare‐Kaseb et al. 2026). Furthermore, FCC accelerates the recovery process for critically ill children, leading to shorter hospitalisation periods (Aljawad et al. 2025; Latour et al. 2024). Consistent implementation of FCC practices is crucial for achieving these positive impacts and ensuring optimal bonding experiences for all families in the PICU (Makino and Ohashi 2017; Salvador et al. 2019).

Some findings were comparable to those of prior FCC studies conducted elsewhere. In Denmark, for example, FCC implementation led to the early discharge of newborns from intensive care, according to (Brødsgaard et al. 2015). Correspondingly, family involvement and support in Italian PICUs were associated with increased family satisfaction, increased weight gain in preterm children, and enhanced recovery (De Bernardo et al. 2017; Raiskila et al. 2016). Families in ICUs can assist with basic care for their sick children, such as cleaning, bathing, feeding, diaper changing, and dressing. The nurses and doctors reported this reduced their workload, enabling them to perform more clinical functions, including resuscitation, monitoring, and drug administration (Latour et al. 2024). Early hospital discharge is, in fact, a sign of high‐quality healthcare delivery (Seniwati, Wanda, and Nurhaeni 2023). These results may point to the essence of FCC practice, and, as a result, health facility administrators, nurses, and doctors should embrace it in ICUs to improve the quality of healthcare delivery.

The implementation of FCC in ICUs has been shown to provide significant holistic value, as evidenced by reduced parental anxiety (Skene et al. 2019). This holistic value goes beyond clinical outcomes to include the emotional, relational, and experiential well‐being of children and their families (Wickramasinghe et al. 2019). The findings of this study confirm earlier research that the intense and often intimidating environment of the ICU, which is characterised by unfamiliar technology, limited access, and the gravity of the child's condition, can exacerbate parental distress and feelings of helplessness (Abukari and Schmollgruber 2025; Bentum et al. 2023; Yakubu et al. 2019). However, when families are actively involved in care, they gain control and capability in caring for critically ill children, reducing anxiety and emotional burden (Abukari and Schmollgruber 2025).

FCC empowers families holistically by providing emotional support, facilitating shared decision‐making, and ensuring open communication (Latour et al. 2024). The findings revealed that when nurses and doctors welcomed families into the care environment and engaged them meaningfully, parents felt respected, heard, and reassured. This is consistent with the relational component of FCC, which acknowledges the therapeutic value of trusting relationships between families, nurses, and doctors (Hodgson et al. 2024; Latour et al. 2024). Furthermore, the participatory model of FCC promotes confidence; as families gain faith in the clinical team's competence, healthcare providers begin to see families as partners rather than passive observers (Latour et al. 2024; Lee 2024). The resulting mutual respect and collaboration can significantly improve family satisfaction and lead to a more compassionate and humane care experience (Hatlie et al. 2020; Kouo et al. 2022).

Incorporating these principles into ICU practice generates a care approach that meets clinical needs and addresses psychological and emotional aspects of care (Espíndola et al. 2024; Wickramasinghe et al. 2019). As a result, FCC becomes a vehicle for quality improvement, not only by improving health outcomes but also by increasing family engagement, reducing emotional trauma, and fostering a culture of empathy and shared responsibility in high‐acuity settings. This study's findings highlight the need for ICUs to adopt a more inclusive, collaborative care delivery model (Latour et al. 2024). When nurses and doctors work in environments that value open family participation, grounded in dignity, respect, and partnership, there is potential for measurable improvements in family satisfaction, therapeutic alliance, and perceived service quality (Chen et al. 2025; Hodgson et al. 2024). This supports the argument that FCC is more than just a philosophy; it is a comprehensive framework for providing ethically sound, emotionally supportive, and clinically effective care (Abukari 2022; Latour et al. 2024).

Earlier research in South Africa advocated for the implementation of FCC in South African PICUs, citing its significant contribution to the development of early parent‐infant bonding and attachment, which has positive long‐term psychosocial outcomes (Maree et al. 2020; Mol et al. 2018). Within the holistic framework of FCC, care's emotional and relational aspects, particularly in high‐stress settings such as PICUs, are just as important as clinical interventions. In this context, one of the most effective FCC practices is facilitating kangaroo care (KC), in which parents maintain skin‐to‐skin contact with critically ill infants. KC promotes physiological stability in newborns, encouraging emotional closeness and strengthens the early attachment process between parent and child (Abukari 2022; Haag et al. 2024).

The involvement of parents in such intimate and nurturing practices within the ICU exemplifies FCC's principles of respect, participation, collaboration, and emotional support (Institute for Patient‐ and Family‐Centred Care 2021). These experiences help bridge the emotional gap created by clinical barriers and medicalised environments, reinforcing the family's central role in the healing process. In line with these principles, the study findings indicate that when families are actively involved in the ICU, particularly through interventions such as KC, their sense of connection, purpose, and confidence in caregiving improves significantly.

Future PICUs should be thoughtfully designed to include dedicated spaces for parent‐infant interaction, further institutionalising the benefits of the FCC and supporting practices such as KC (Maree et al. 2017; Staniszewska et al. 2012). The addition of KC rooms would not only increase physical closeness but would also symbolically reinforce the family's integral role in care, resulting in a more humane and relationally attuned ICU environment (Axelin and Raiskila 2020; Tandberg et al. 2018). By doing so, healthcare systems can move towards a model that recognises and nurtures the therapeutic power of family presence, eventually improving both short‐ and long‐term health and relational outcomes for infants and their families (de Mingo‐Fernández et al. 2021; Jakab et al. 2019).

The implementation of FCC in intensive care units (ICUs) benefits not only patients and families but also the overall functioning and satisfaction of the healthcare team (Aljawad et al. 2025; Latour et al. 2024). FCC improves communication channels between healthcare providers and families by facilitating the timely, transparent flow of information, which is critical in high‐acuity settings where clinical decisions change rapidly (Benzies 2016; Massa et al. 2021). Conceivably, when families are actively involved and kept informed, they are better equipped to make meaningful contributions to care decisions, reducing confusion, anxiety, and potential conflicts.

According to (Ambardekar et al. 2013), the American Academy of Paediatrics has stated that FCC integration can reduce job‐related fatigue among healthcare providers, improve medical record accuracy and completeness, and increase overall job satisfaction. These advantages are inextricably linked to the relational and operational efficiencies that FCC fosters with families. For example, taking on supportive roles, including assisting with basic caregiving tasks such as diaper changing, allows nurses and doctors to devote more time to critical interventions, thereby lightening their workload and improving workflow (Abukari 2022; Gunnlaugsdóttir et al. 2024).

Furthermore, the FCC fosters mutual trust among nurses, doctors, and families (Simpson‐Collins et al. 2024). In systems with limited or inconsistent family involvement, misunderstandings about the child's condition and treatment processes can arise, potentially leading to dissatisfaction, mistrust, or even legal disputes. In contrast, when families are involved as care partners, they better understand the medical context, feel heard and respected, and are more likely to collaborate constructively with healthcare teams (Dijkstra et al. 2025; Seniwati, Rustina, et al. 2023). The respondent's recognition of FCC's value in ICU protocols indicates a broader understanding of FCC's holistic value. Consequently, FCC strengthens a therapeutic alliance by emphasising communication, relational trust, and shared responsibility, thereby promoting ethical care delivery, protecting institutional integrity, and improving clinical outcomes for children and families.

### 5.1 Implications

Using a mixed‐methods approach with factor analysis, this study's findings corroborate earlier studies on FCC in this context, strengthening evidence of the positive impact of FCC application in ICUs. Our study highlights the critical need for the acceptance, integration, and implementation of FCC across paediatric care settings, particularly in PICUs. FCC integration into PICUs could improve quality health outcomes, particularly for children and their families. We propose that nurses and doctors should undergo additional in‐service training on FCC integration into the PICU setting. Additionally, heads of PICUs could modify the set‐up to create space for easy FCC integration.

In PICUs, FCC has significant clinical and psychosocial benefits. Its implementation could improve parent–child bonding, lower parental stress and anxiety, and increase family involvement in decision‐making, all of which contribute to better child health outcomes and recovery timelines (Chen et al. 2025; Gunnlaugsdóttir et al. 2024). Furthermore, the FCC could address the emotional and ethical aspects of paediatric critical care, promoting trust, dignity, and shared responsibility among families and healthcare teams. FCC may improve quality of care and safety in high‐acuity settings by bridging communication gaps and strengthening relationships (Abukari 2022; Hodgson et al. 2024).

The researchers proposed integrating FCC concepts, communication strategies, and ethical frameworks into nursing curricula and ongoing professional development programmes (Çınar Özbay et al. 2023). For instance, the Nursing and Midwifery Council (NMC), in partnership with academic institutions and regulatory bodies such as the Ghana Health Service (GHS), Ghana College of Nurses and Midwives, Health Facility Regulatory Authority (HeFRA), and the Ministry of Health (MoH), should prioritise the development of structured FCC training modules. These modules would provide healthcare professionals, including nurses and doctors, with the necessary skills to implement FCC in real‐world settings, particularly in the demanding environments of the ICU.

Furthermore, from a healthcare policy standpoint, there is an urgent need to develop regulatory guidelines and infrastructure standards to support FCC implementation. For example, designing PICUs with dedicated family spaces, privacy for special cases, and protocols for active family participation in clinical discussions would help institutionalise the FCC principles and move them from policy rhetoric to routine practice (Staniszewska et al. 2012; Tronstad et al. 2023).

Importantly, FCC's holistic value lies in its ability to improve patient outcomes while reducing staff burnout and increasing organisational efficiency (Havaei et al. 2020). In resource‐constrained environments, the FCC can catalyse the redistribution of caregiving responsibilities, reducing the strain on overburdened ICU nurses and doctors. When families can contribute to non‐clinical aspects of care, such as hygiene or comfort measures, nurses and doctors can focus on more complex procedures, improving workflow and quality of care (Dijkstra et al. 2025; Gunnlaugsdóttir et al. 2024). In the end, these findings confirm the existing literature on FCC and could contribute to a context‐specific understanding of it in ICUs.

### 5.2 Limitations

The findings presented in this research are subject to some limitations and should be interpreted cautiously. The data came from two PICUs, which may not be sufficiently representative for generalisation, although the data were triangulated to address this limitation. It is possible that including regional ICUs could have increased data variability. We did not perform a subgroup analysis of the perceived value of FCC because the dataset was too small for stratification. The differences in role and professional training among the included professionals could have led to variations in the holistic value of FCC; as such, future research may explore this angle.

Another limitation of the study was that the quantitative stage also excluded families; their inclusion could have added data variability. Despite the high response rate (> 95%) in the quantitative phase, family perspectives were not included. The family's exclusion in the quantitative phases was due to their greater inclusion in the qualitative phase. Additionally, the quantitative phase was designed to validate the perceived holistic value of FCC from experts' perspectives. Future research may apply confirmatory factor analysis to isolate and confirm the holistic value of FCC in our setting and in other contexts with similar characteristics, thereby enabling generalisability.

## 6 Conclusion

We triangulated and mapped multiple sources of data to demonstrate the holistic value of FCC in a low‐resource PICU setting. Our findings corroborate previous studies on FCC and underscore the context‐specific value of FCC in PICUs, particularly in promoting communication and patient teaching, which could reduce clinical workload and promote patient recovery. The participants perceived that FCC improved parent–child bonding and attachment, reduced parental anxiety, built trust in healthcare teams, and promoted family satisfaction with delivery of care.

Given the perceived holistic value of FCC demonstrated in this study, we recommend that policymakers, training institutions, and hospital administrators prioritise in‐service training and workshops for healthcare professionals and families to increase staff members' capacity in FCC principles and practices. Furthermore, future research should corroborate our findings by using diverse neonatal and paediatric settings. To improve quality paediatric and child health outcomes, unit heads, nurses, and doctors should consider modifications of the ICU set‐up to create space, which could maximise the integration of FCC in practice, particularly in NICUs and PICUs.

## Author Contributions

Study design: Alhassan Sibdow Abukari, Shelley Schmollgruber, and Emmanuel Kwame Korsah.; Data collection: Alhassan Sibdow Abukari; Data analysis: Alhassan Sibdow Abukari, Shelley Schmollgruber, Emmanuel Kwame Korsah. Manuscript preparation: Alhassan Sibdow Abukari, Shelley Schmollgruber, Emmanuel Kwame Korsah.

## Funding

The authors have nothing to report.

## Disclosure

The authors acknowledge that Dr. LeeAnne Masilela, a biomedical statistician from the University of the Witwatersrand, Faculty of Health Sciences, Public Health, holding a MSc (Epidemiology) and MBBCH analysed the data.

## Ethics Statement

We obtained ethical clearance from the human research ethics committees of the two local facilities in Ghana (TTH/R&D/053 and STC/IRB/000145/2019) and the University of the Witwatersrand, South Africa (M190980). The initial ethics was approved in 2019, and an extension was granted in 2022. Additionally, we provided an information sheet to all participants, and those who agreed to participate signed a voluntary consent form before the study. Additional consent for voice recording was voluntarily obtained from the participants. We used the introductory letters from the ethics committee to access the department and the facilities for data collection. Also, we followed all ethical guidelines on confidentiality, autonomy, and voluntary participation as enshrined in the Helsinki Declaration.

## Consent

All the participants gave their consent for the information to be published.

## Conflicts of Interest

The authors declare no conflicts of interest.

## Data Availability

The data that support the findings of this study are available on request from the corresponding author. The data are not publicly available due to privacy or ethical restrictions.
